# Bidirectional Associations Between Self-Reported Gaming Disorder and Adult Attention Deficit Hyperactivity Disorder: Evidence From a Sample of Young Swiss Men

**DOI:** 10.3389/fpsyt.2018.00649

**Published:** 2018-12-11

**Authors:** Simon Marmet, Joseph Studer, Véronique S. Grazioli, Gerhard Gmel

**Affiliations:** ^1^Alcohol Treatment Centre, Lausanne University Hospital/CHUV, Lausanne, Switzerland; ^2^Addiction Switzerland, Lausanne, Switzerland; ^3^Centre for Addiction and Mental Health, Toronto, ON, Canada; ^4^Department of Health and Social Sciences, University of the West of England, Frenchay, Bristol, United Kingdom

**Keywords:** video gaming, gaming disorder, attention deficit hyperactivity disorder, early adulthood, autoregressive cross-lagged modelling, Switzerland

## Abstract

**Background:** Gaming disorder (GD) has been shown to co-occur with attention deficit hyperactivity disorder (ADHD), yet few studies to date have investigated their longitudinal associations.

**Method:** The sample included 5,067 young Swiss men (mean age was 20 years at wave 1 and 25 years at wave 3). Measures were the Game Addiction Scale and the Adult ADHD Self-Report Scale (6-item screener). Longitudinal associations were tested using autoregressive cross-lagged models for binary measures of GD and ADHD, as well as continuous measures for GD score and ADHD subscales of inattention and hyperactivity.

**Results:** ADHD at age 20 increased the risk for GD at age 25 (probit = 0.066 [0.023, 0.109]; *p* = 0.003). GD at age 20 also increased the risk for ADHD at wave 3 (probit = 0.058 [0.013, 0.102]; *p* = 0.011). Only the ADHD inattention subscale showed a bidirectional longitudinal relationship with the GD score (standardized Beta from inattention at age 20 to GD score at age 25: 0.090 [0.056, 0.124]; *p* < 0.001; from GD score at age 20 to inattention at age 25: 0.044 [0.016, 0.071]; *p* = 0.002), whereas associations between the hyperactivity subscale and GD were not significant.

**Discussion:** GD had bidirectional longitudinal associations with ADHD, in that ADHD increased the risk for GD and GD increased the risk for ADHD, and they may reinforce each other. These associations may be linked more to the inattention ADHD component than to the hyperactivity ADHD component. Individuals with ADHD or GD should be screened for the other disorder, and preventive measures for GD should be evaluated in individuals with ADHD.

## Introduction

### Gaming Disorder

Video gaming is a widespread activity among young men. Although gaming is an unproblematic leisure activity like many others for most people (1), it does cause problems for some, eventually resulting in a gaming disorder (GD), for which prevalence estimates in European adolescent nationally representative general population surveys range from about 1 to 5% (2–4). Prevalence rates may be higher in Asian countries(4, 5). GD is more frequent in younger age groups and men (3, 4, 6). GD has been defined as an excessive and compulsive use of video games resulting in social and/or emotional problems (7). It has also been associated with several mental health problems such as major depression, attention deficit hyperactivity disorder (ADHD), anxiety, and social phobia/anxiety (8, 9). There is some controversy about whether GD should be labelled as a behavioural (i.e., non-substance) addiction/disorder (10–12). It is not included as such in the current fifth edition of the Diagnostic and Statistical Manual of Mental Disorders (DSM-5) (13). However, a GD subtype, namely internet gaming disorder, is under consideration for inclusion as a psychiatric disorder in the DSM-5. GD is not included in the current International Classification of Diseases (ICD-10) either, but it will be included as “gaming disorder” in the upcoming ICD-11 (14), without the prefix “internet,” unlike in DSM-5. Different terms are in use for “gaming disorder,” notably “gaming addiction” or “problematic gambling.” The term “gaming disorder” is used here because its use in the DSM-5 and ICD-11 is likely to make it the most popular term in the future. The present study investigates longitudinally how GD is associated with another common disorder in young men, namely ADHD.

### Attention Deficit Hyperactivity Disorder (ADHD)

ADHD is classified as a neurodevelopmental disorder. It is characterised by two components: inattention (e.g., often distracted) and hyperactivity (e.g., an urge to move) (13). Prevalence rates of ADHD in school-age children range from about 5 to 7% (15). However, studies have shown that the symptoms of ADHD may persist into adulthood in about one to two thirds of cases and that ADHD may affect as many as 2.5 to 5% of the general population (15). Untreated, ADHD is associated with behavioural, emotional, social, academic, and vocational problems (15). Furthermore, ADHD was also found to be related to mental health problems and addictive disorders (16–20), as well as with lower life satisfaction (21).

### ADHD and Gaming

There has been relatively little research about the link between GD and ADHD. This is partly because before the DSM-5 included internet GD as a condition for further study, in 2013, internet GD was often studied together with internet addiction, and only afterwards as an independent condition (22). In a recent review, González-Bueso and Santamaría (8) identified eight studies that investigated the link between internet gaming disorder and ADHD specifically, of which seven (85%) reported a significant association, four of these reported a large effect size (OR ≥ 4.25). The only longitudinal study (23) included in their review reported no association between GD and ADHD. An earlier review also found these associations (22). A more recent longitudinal study of a sample of adolescents (with adolescents at a high risk of GD being oversampled), not included in the above reviews, found that parent-reported hyperactivity/inattention predicted self-reported internet GD 1 year later, but self-reported internet GD did not significantly predict parent-reported hyperactivity/inattention 1 year later (24).

Regarding associations with ADHD's inattention and hyperactivity subscales, another recent study reported that attention problems (only the inattention subscale was measured) in adolescents predicted internet GD 1 year later (25). A cross-sectional study of 205 adults also found that GD was only linked to ADHD's inattention subscale and not its hyperactivity subscale (26). In contrast, a study in young children (27) found that the inattention subscale was more strongly associated with GD in girls, whereas the hyperactivity subscale was more strongly associated with GD in boys.

Several theories have been proposed for the link between ADHD and GD. For example, the “optimal stimulation model” proposes that individuals with ADHD have a higher threshold for reaching an agreeable level of arousal, and the rapid visual and acoustical stimulations in computer games requiring fast motor responses may be one way to reach this level (27). Another theory, the“delay aversion theory” suggests that individuals with ADHD prefer smaller immediate rewards over larger delayed rewards, and computer games may provide such immediate and continuous rewards (27). Furthermore, individuals with ADHD may suffer from a reward deficiency syndrome with deficiencies in dopamine neurotransmission: video games resulting in significant dopamine release may, therefore, be a way to cope with this reward deficiency (28). The same mechanism may also explain the high comorbidity between ADHD and substance use disorders (SUDs). Panagiotidi (26) also proposed that gaming may improve visual attention, which tends to be impaired in individuals with ADHD, who may therefore be gaming as a means to counter this deficit. Indeed, a recent review (3) found an association between video gaming and visual attention, however, this association was rather small and a causal relationship has yet to be established. However, while some theories explaining the link between GD and ADHD exist, there is currently a lack of empirical evidence supporting these theories, and it remains possible that there is no causal link between ADHD and GD.

Most explanations and research have focussed on how ADHD leads to GD, although some explanations for a relationship in the other direction have also been proposed. Notably, ADHD symptoms may make gaming more attractive, whereas increased gaming may, in turn, exacerbate ADHD symptoms “by providing an activity that continuously reinforces the exact disinhibition, quick responsiveness, need for immediate reward, and inattention that are areas of concern” (29). A study among children and adolescents (30) showed that greater television and video game exposure (hours spent playing or watching television) was associated with greater attention problems 13 months later, even when controlled for earlier attention problems. Another study (31) even found bidirectional associations between video gaming exposure and attention problems, suggesting that children with attention problems may spend more time playing, which may increase their subsequent attention problems. The authors also suggested that electronic screen media, e.g., video games, especially those involving violence, may be highly exciting and, over time, increase an individual's threshold for a desired level of stimulation, which may then lead to problems focusing on less exciting activities like work or study (the “excitement hypothesis”) (31). An alternative hypothesis, the “displacement hypothesis,” assumes that individuals spending a lot of time playing games spend less time with cognitively and physically more appropriate activities that may improve their ability to focus (27, 31).

### Aims

This study aimed to re-examine the association between GD and ADHD in a longitudinal sample of young Swiss men. We first investigated whether our data confirmed cross-sectional associations between GD and ADHD and the ADHD subscales of inattention and hyperactivity. In a second step, we tested the longitudinal associations between GD and ADHD using an autoregressive cross-lagged (ARCL) model. The model examined whether ADHD at age 20 was associated with GD at age 25, whether GD at age 20 was associated with ADHD at 25, or whether there were bidirectional associations between GD and ADHD. We also tested GD for longitudinal associations with ADHD's inattention and hyperactivity subscales. In a third step, we tested whether participants with ADHD and GD at wave 1 (at about 20 years old) had worse outcomes with both of those disorders at wave 3 (about 25 years old) than participants with GD only or ADHD only, as well as several other outcomes potentially associated with ADHD or GD, namely major depression, mental health, life satisfaction, and poor performance at work or school.

## Methods

### Sample

The sample stems from the Cohort Study on Substance Use Risk Factors (C-SURF; www.c-surf.ch). This study follows a large sample of young Swiss men recruited in their late adolescence to their adulthood, with measurement points at the age of about 20, 21, and 25 years, with more measurement waves being in planning. The main aim of the study is to evaluate patterns, trajectories, and associated risk or protective factors of substance use and non-substance related behaviours in these young men (32, 33).

Enrolment for the baseline assessment took place between August 2010 and November 2011 in three of six national Swiss army recruitment centres, located in Lausanne, Windisch and Mels (covering 21 out of 26 Swiss cantons), during the recruitment procedure for military service. These procedures are mandatory for all young Swiss men at about the age of 20, therefore the sampling at this occasion has the advantage of covering most of young men of that cohort. Responses to questionnaires were independent of army procedures as individuals responded privately at home and confidentiality from the army was ensured. Participants could choose between paper questionnaires per mail or online questionnaires which were accessible by a link sent per e-mail. A total of 13,237 of young men have been asked to participate in the study, and 7,556 finally gave their written consent to participate in the study, of which 5,987 returned the baseline questionnaire (wave 1) and 5,516 returned the second follow-up questionnaire (wave 3) between April 2016 and March 2018. To increase response rates, participants who did not answer the questionnaire after standard reminders were encouraged by trained interviewers via phone calls to participate (33).

The present study includes all 5,125 (85.6% retention rate) participants who responded to the baseline and second follow-up questionnaires. Of those, 58 (1.1%) participants with missing values for GD or ADHD in waves 1 or 3 were excluded, leaving 5,067 participants included in our present analysis. Participants received vouchers (50 CHF per questionnaire) as compensation for their efforts. Data from wave 2 were not used (except for imputing missing values, see statistical analysis section) because the measure for ADHD was only included in waves 1 and 3. The research protocol was approved by the Human Research Ethics Committee of the Canton Vaud (Protocol No. 15/07).

### Measures

#### Gaming Disorder and ADHD

##### Gaming disorder

Gaming disorder (GD, last 6 months) was measured using the Game Addiction Scale (GAS) (7), which was translated into German and French for this study. The scale consists of seven Likert-type items with five response options ranging from 0 (*never*) to 4 (*very often*), and participants who responded to at least three items with a score of at least 2 (*sometimes*) were defined as presenting GD, as suggested by Lemmens and Valkenburg (7). Additionally, a continuous score as the sum of the seven items was used (ranging from 0 to 28). The wording of the GAS changed slightly between wave 1 and wave 3. In wave 1, the wording included, in addition to gaming, time spent on the internet (e.g., “Have you felt upset when you were unable to play *or to spend time on the internet?*”; italic part was added and differed from the original wording of the GAS). This was done, because at the time when the questionnaire for wave 1 was developed, it was thought that lots of games involve internet activities, and that GD may be impossible without spending time in the internet (online games). After the DSM-5 (13), released in 2013, included internet GD as a condition for further study, it became evident that gaming should subsequently be measured distinctly and not be mixed with time spent on the internet, and the original Game Addiction Scale (without adding reference to the internet in the wording of the questions) was therefore used in wave 3. To account for the differences in wording of the GAS in wave 1 and wave 3, to improve comparability across waves, and to reduce false positives, the GD scores of participants who did not play games at least weekly (and therefore may have a GAS score due to non-gaming related Internet use) were set to 0 in both waves. Cronbach's Alpha for the GAS scale was 0.895 in wave 1 and 0.868 in wave 3.

##### Adult attention deficit hyperactivity disorder

Adult attention deficit hyperactivity disorder (ADHD, last 12 months) was measured using the six-item screener version of the Adult ADHD Self-Report Scale (ASRS-v1.1) (34) developed by the World Health Organization (WHO) and based on the DSM-IV diagnostic criteria (35). Four items assessed the ADHD inattention subscale and two items assessed its hyperactivity subscale (see **Table 2**). Response options were on a five-point Likert-type scale ranging from 0 (*never*) to 4 (*very often*). For building a binary measure of ADHD, items were dichotomised—at least 2 (*sometimes*) for the first three items and at least 3 (*often*) for the last three items—and ADHD was defined as the presence of at least 4 symptoms as suggested by the authors of the scale (34). For analysis involving the continuous ADHD subscales of inattention and hyperactivity, the mean of the Likert-scale items (with values ranging from 0 to 4) was calculated. Cronbach's Alpha for the ADHD scale was 0.798 in wave 1 and 0.778 in wave 3.

#### Substance Use Disorder Scales

##### Alcohol use disorder

Alcohol use disorder (AUD, last 12 months) was measured using 12 items for the 11 DSM-5 criteria (13, 36, 37) for AUD in a yes/no format. The DSM-5 moderate (4+) cut-off was used to define AUD. Cronbach's Alpha for the AUD scale was 0.729 in wave 1 and 0.696 in wave 3.

##### Cannabis use disorder

Cannabis use disorder (last 12 months) was measured using the revised version of the Cannabis Use Disorder Identification Test [CUDIT-R; (38), based on (39)]. The test consists of 8 five-point Likert-type items ranging from 0 (*never*) to 4 (*daily or almost daily*), a measure of the frequency of cannabis use ranging from 1 (monthly or less often) to 4 (four or more times per week), and one item with two response options, 0 (smoking cannabis for fun) or 4 (smoking cannabis out of habit). A cut-off of 8 out of 40 possible points was used to define cannabis use disorder. Cronbach's Alpha for the cannabis use disorder scale was 0.894 in wave 1 and 0.906 in wave 3.

##### Tobacco use disorder

Tobacco use disorder (last 12 months) was assessed using six items from the Fagerström Test for Nicotine Dependence (FTND (40). A cut-off of 3 out of 10 possible points was used to define tobacco use disorder. Cronbach's Alpha for the tobacco use disorder scale was 0.719 in wave 1 and 0.702 in wave 3.

#### Major Depression and Mental Health

##### Symptoms of major depression

Symptoms of major depression in the last 2 weeks were measured using the WHO's Major Depressive Inventory (41), consisting of 12 six-point Likert-type statements measuring 10 criteria and ranging from 0 (*never*) to 5 (*always*); two criteria were assessed using two statements each, with only the highest value of the two statements being used for the sum score. The sum of the criteria scores, ranging from 0 to 50, was used in this analysis. Cronbach's Alpha for the major depression scale was 0.889 in wave 1 and 0.888 in wave 3.

##### Mental health

Mental health was assessed using the Medical Outcomes Study 12-Item Short Form Survey Instrument, v2 (SF-12) (42). The mental component summaries were linearly transformed into norm-based scores (mean = 50; *SD* = 10). Cronbach's Alpha for the SF-12 mental health scale was 0.772 in wave 1 and 0.790 in wave 3.

#### Life Satisfaction and Poor Performance at Work/School

##### Life satisfaction

Life satisfaction was measured using the Satisfaction with Life Scale (43), consisting of five items with seven response options ranging from 1 (*strongly disagree*) to 7 (*strongly agree*). The sum of the items (ranging from 5 to 35) was calculated for the analysis. Cronbach's Alpha for the life satisfaction scale was 0.772 in wave 3. Life satisfaction was not measured in wave 1.

##### Poor performance at work/school

Poor performance at work/school was measured in wave 1 and wave 3 using a single question asking participants whether they had performed poorly at school or work, or got behind with work, in the last 12 months. Response options were from never to 10 or more times. This question was adapted from the ESPAD survey (44).

For all the scales used, missing values on single items were replaced by the scale mean. If more than 20% of the scale's items were missing, the scale was considered to be missing.

### Statistical Analysis

Descriptive statistics were computed, and changes in prevalence rates of GD and ADHD between baseline (wave 1) and the second follow-up (wave 3) were tested using McNemar chi-square tests. Cross-sectional differences between participants with and without GD were tested using logistic regressions. All regressions were adjusted for age and linguistic region. Descriptive statistics and data preparation were done using SPSS 25. For testing longitudinal associations between GD and ADHD, ARCL models were estimated using MPLUS 8.0 (45). ARCLs are a form of structural-equation modelling often used for describing developmental processes between two (or more) constructs across multiple time points [for an overview, see (46)]. Our main interests were the cross-lagged paths representing the longitudinal effect of GD at age 20 on ADHD at age 25, and of ADHD at age 20 on GD at age 25, taking into account the autocorrelation of the same construct across time points and the cross-sectional correlation between different constructs at the same time point. For the binary measures of GD and ADHD, the ARCL was estimated using the weighted least square mean and variance adjusted (WLSMV) estimator, which returns for binary variables probit regression coefficients. The WLSMV estimator allows the correlation between the variables at the same time point to be modelled directly. For additional ease of interpretation, probit coefficients were transformed into OR-equivalents. ORs can be approximated by multiplying probit coefficients by the standard deviation of the logistic distribution [$\sqrt{\left(\Pi^{2}/3\right)}$ = 1.81] and then using the exponential function of the resulting coefficient (47). For the ARCL between the continuous GD score and the ADHD inattention and hyperactivity subscales, we used the Robust Maximum-Likelihood estimator (MLR), which is robust to skewness in the outcome variables. In a third step, we investigated whether participants with both GD and ADHD at wave 1 had a worse situation regarding GD, ADHD, major depression, mental health, life satisfaction, and poor performance at work or school at wave 3 than did participants with neither GD nor ADHD, or with GD alone or ADHD alone. Differences between these groups were also tested using logistic regressions for binary outcomes, with ordinal regressions for ordinal outcomes (poor performance at work or school) and with linear regression for continuous outcomes (scale scores). Regressions for major depression, mental health, and poor performance at work or school were adjusted for their respective baseline values (at age 20). Baseline values were not available for life satisfaction.

Given that SUDs are associated with ADHD, e.g., (19), as well as with GD (1), all our longitudinal analyses were adjusted by the continuous scores of the alcohol, tobacco and cannabis use disorder scales at wave 1 to control for the effect of SUD's co-occurring with GD or ADHD at wave 1 on GD and/or ADHD at wave 3. Because our interest in these analyses was in the longitudinal effect of GD and ADHD, the longitudinal analyses were not adjusted for SUD at wave 3. Also, SUD's at wave 3 may be in part a consequence of GD and ADHD at wave 1, and adjusting for them may therefore remove a part of the true effect of GA or ADHD at wave 1 on GD and ADHD at wave 3. Missing values on these SUD scales were imputed for 264 cases in wave 1 and 49 cases in wave 3, using multiple imputations in MPLUS 8.0 in a Bayesian framework, creating 20 imputed data sets using the SUD scales as well as use measures for the three substances in all three waves plus age and language. Overall, the impact of SUDs on the associations between GD and ADHD was small, and we therefore only show analyses adjusted by SUDs in the tables and figures.

## Results

### Cross-Sectional Associations

Table 1 shows descriptive results and prevalence rates of GD, ADHD, and SUDs. Prevalence of GD decreased from 8.8% in wave 1 to 6.3% in wave 3 [McNemar test $\chi_{\left(1\right)}^{2}$ = 29.81; *p* < 0.001]. Prevalence of ADHD increased from 5.7% in wave 1 to 7.6% in wave 3 [McNemar test $\chi_{\left(1\right)}^{2}$ = 18.68; *p* < 0.001]. Cross-sectionally, ADHD was more frequent in participants with GD than without GD, in both waves, with an Odds Ratio (OR) of 3.21 [2.39, 4.32] for wave 1 and 2.56 [1.86, 3.52] for wave 3. SUDs were not significantly associated with GD in wave 1, yet SUDs were significantly more frequent in participants with GD than without GD in wave 3. Accordingly, adjusting for SUDs only marginally changed the association between ADHD and GD in wave 1, but reduced this association in wave 3 (from OR = 2.56 to OR = 2.08). The mean scores of each of the six ADHD items were higher in participants with GD at waves 1 and 3, although this was not significantly higher for the second item of the ADHD hyperactivity subscale (“driven by a motor”; Table 2). Both the inattention and hyperactivity subscale scores were cross-sectionally associated with GD in waves 1 and 3, however, differences between participants with and without GD were more pronounced for the inattention subscale (see Table 2). When both subscales were entered into a regression model with GD as the outcome, only inattention was significantly associated with GD (Table 2) in both waves.

**Table 1:** Sample statistics and cross-sectional associations between gaming disorder and ADHD.

	**Wave 1 (age 20)**	**Wave 3 (age 25)**
*n*	5,067	5,067
Mean age	19.97	25.44
*SD* age	1.22	1.25
German-speaking % (*n*)	44.0% (2,230)	44.0% (2,230)
French-speaking % (*n*)	56.0% (2,837)	56.0% (2,837)
**PREVALENCE OF GAMING DISORDER BY ADHD STATUS**		
Total sample % (*n*)	8.8% (448)	6.3% (317)
No ADHD % (*n*)	8.0% (383)	5.7% (265)
ADHD % (*n*)	22.4% (65)	13.5% (52)
Odd's ratio [95%CI] for GD on ADHD	**3.21 [2.39, 4.32]**	**2.56 [1.86, 3.52]**
Adjusted for substance use disorders	**3.27 [2.41, 4.44]**	**2.10 [1.51, 2.93]**
**PREVALENCE OF ADHD BY GAMING DISORDER STATUS**		
Total sample % (*n*)	5.7% (290)	7.6% (385)
No GD % (*n*)	4.9% (225)	7.0% (333)
GD % (*n*)	14.5% (65)	16.4% (52)
Odd's Ratio [95%CI] for ADHD on GD (ref.)	**3.21 [2.39, 4.32]**	**2.56 [1.86, 3.52]**
Adjusted for substance use disorders	**3.33 [2.45, 4.51]**	**2.08 [1.49, 2.91]**
**ODD'S RATIOS [95%CI] FOR GD ON SUBSTANCE USE DISORDERS**		
Alcohol use disorder (ref. no disorder)	1.22 [0.90, 1.66]	**1.77 [1.27, 2.47]**
Tobacco use disorder (ref. no disorder)	1.14 [0.88, 1.49]	**2.13 [1.64, 2.76]**
Cannabis use disorder (ref. no disorder)	0.84 [0.57, 1.24]	**2.41 [1.74, 3.34]**

*ADHD, attention deficit hyperactivity disorder; GD, gaming disorder. All regressions were adjusted for age and language. Significant (p < 0.05) coefficients from logistic regressions are in bold*.

**Table 2:** Differences in means of individual ADHD items and ADHD subscales among participants with and without gaming disorder.

	**Wave 1 (age 20)**			**Wave 3 (age 25)**		

	**Mean by group**			**Mean by group**	
	**No GD**	**GD**	**OR [95% CI]**	**No GD**	**GD**	**OR [95% CI]**
	**(*****n*** **=** **4,619)**	**(*****n*** **=** **448)**	**(ref. no GD)**	**(*****n*** **=** **4,750)**	**(*****n*** **=** **317)**	**(ref. no GD)**
**INATTENTION ADHD ITEMS (VALUES FROM 0 TO 4)**						
How often do you have trouble wrapping up the final details of a project, once the challenging parts have been done?	0.77	1.04	**1.36 [1.23, 1.51]**	1.04	1.38	**1.39 [1.25, 1.55]**
How often do you have difficulties getting things in order when you have to do a task that requires organization?	0.70	1.12	**1.56 [1.41, 1.72]**	0.87	1.35	**1.66 [1.48, 1.85]**
How often do you have problems remembering appointments or obligations?	0.71	1.07	**1.50 [1.36, 1.65]**	0.90	1.35	**1.58 [1.41, 1.76]**
When you are working on something that requires a lot of thinking, how often do you postpone or avoid the task?	1.03	1.60	**1.55 [1.43, 1.69]**	1.19	1.71	**1.57 [1.41, 1.74]**
**HYPERACTIVITY ADHD ITEMS (VALUES FROM 0 TO 4)**						
How often do you fidget or squirm with your hands or feet when you have to sit down for a long time?	1.29	1.67	**1.24 [1.15, 1.34]**	1.52	1.92	**1.28 [1.17, 1.40]**
How often do you feel overly active and compelled to do things, like you were driven by a motor?	0.95	1.05	1.09 [1.00, 1.19]	1.16	1.21	1.04 [0.93, 1.16]
**MEANS FOR INATTENTION AND HYPERACTIVITY SUBSCALES**						
Inattention scale mean (0–4)	0.80	1.21	**1.91 [1.69, 2.16]**	1.00	1.45	**2.06 [1.79, 2.38]**
Inattention adjusted for hyperactivity			**1.93 [1.69, 2.21]**			**2.10 [1.79, 2.46]**
Hyperactivity scale mean (0–4)	1.12	1.36	**1.25 [1.13, 1.37]**	1.34	1.56	**1.25 [1.11, 1.40]**
Hyperactivity adjusted for inattention			0.99 [0.89, 1.10]			0.96 [0.84, 1.10]
**ADJUSTED FOR SUD SCORES**						
Inattention scale mean (0–4)			**1.98 [1.74, 2.25]**			**1.92 [1.66, 2.23]**
Inattention adjusted for hyperactivity			**2.00 [1.74, 2.31]**			**1.99 [1.69, 2.34]**
Hyperactivity scale mean (0–4)			**1.24 [1.12, 1.36]**			**1.17 [1.04, 1.32]**
Hyperactivity adjusted for inattention			0.99 [0.88, 1.10]			0.93 [0.81, 1.07]

*GD, gaming disorder. Item scores ranged from never (coded 0) to very often (coded 4). SUD, substance use disorder. ADHD, attention deficit hyperactivity disorder. Logistic regressions are with GD as the outcome, adjusted for age and language. Significant (p < .05) coefficients are in bold*.

### Longitudinal Associations

**Figure 1:**
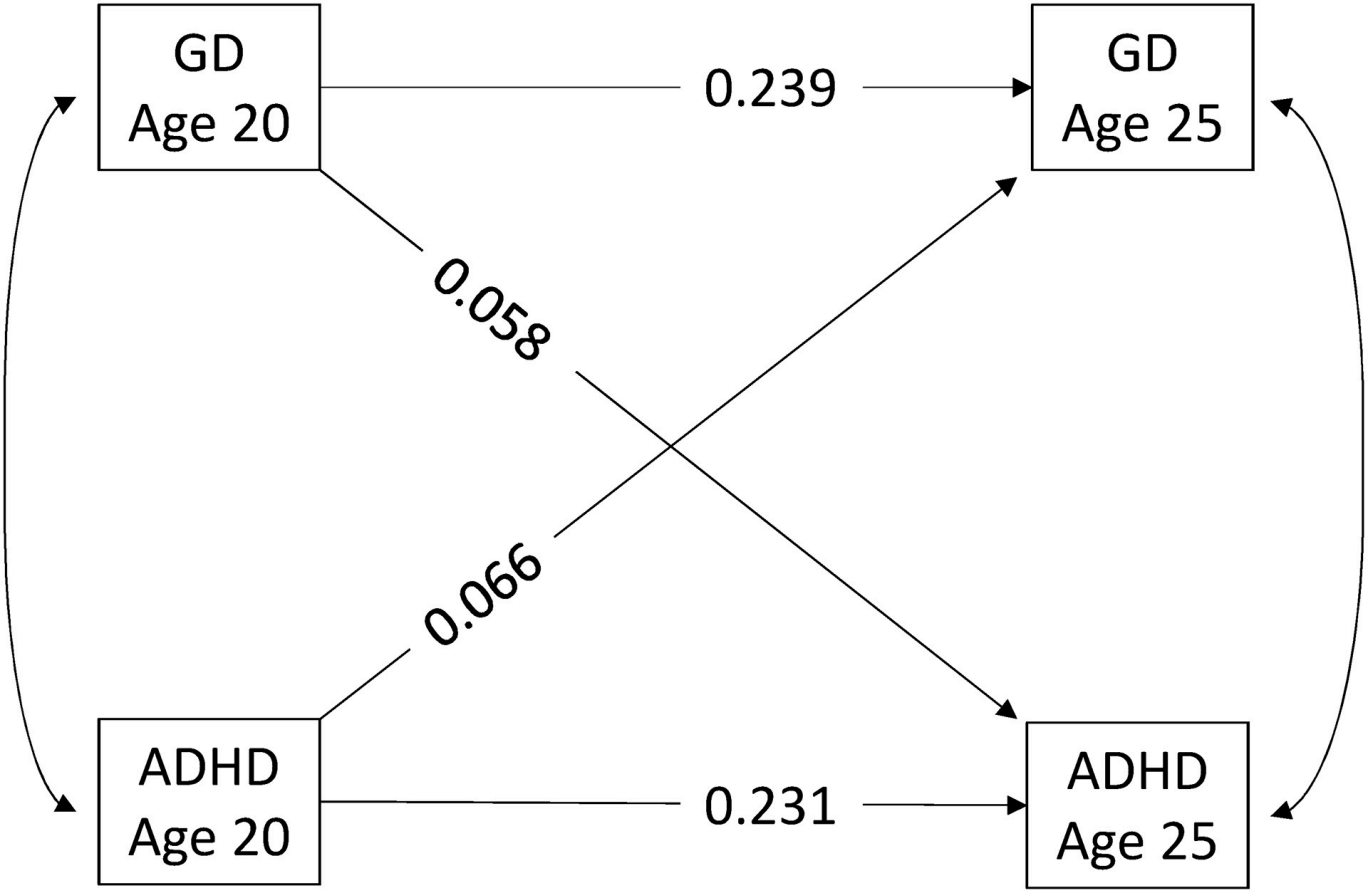
Autoregressive cross-lagged model between binary measures for gaming disorder and ADHD. All the paths shown are significant at the *p* < .05 level. WLSMV was the estimator used. Coefficients are standardised probit. Adjusted for age, language, and substance use disorders at wave 1. ADHD, attention deficit hyperactivity disorder.

Participants with GD at wave 1 were more likely to show ADHD at wave 3, and participants with ADHD at wave 1 were more likely to show GD at wave 3 (Table 3). These associations were tested using an ARCL model (Figure 1), which showed that GD and ADHD had significant bidirectional longitudinal associations, even when considering auto-correlation of the same measure across time and correlation between GD and ADHD at the same time point. The coefficient for ADHD at wave 1 on GD at wave 3 was similar (standardised probit = 0.066 [0.023, 0.109]; *p* = 0.003; corresponding to an OR of 1.72) to the coefficient for GD at wave 1 on ADHD at wave 3 (standardised probit = 0.058 [0.013, 0.102]; *p* = 0.011; corresponding to an OR of 1.47). Adjustments for SUD had only a minor impact on the cross-lagged paths (coefficients unadjusted for SUD were 0.078 and 0.057, results not shown).

**Table 3:** Prevalence and scores of gaming disorder and ADHD in wave 3 as a function of gaming disorder and ADHD status at wave 1.

**GD and ADHD at wave 1 (age 20)**	**Outcomes at wave 3 (age 25)**										

	***n***	**GD (%)**	**OR [95% CI]**	**GD score**	***b*** **[95% CI]**	**ADHD (%)**	**OR [95% CI]**	**ADHD score**	***b*** **[95% CI]**	**ADHD and GD (%)**	**OR [95% CI]**
No GD	4619	4.7	ref.	2.29	ref.	6.8	ref.	6.70	ref.	0.8	ref.
GD	448	22.1	**5.71 [4.39, 7.43]**	6.53	**4.23 [3.64, 4.81]**	15.6	**1.83 [1.35, 2.47]**	8.11	**1.38 [0.93, 1.82]**	3.8	**5.13 [2.84, 9.28]**
No ADHD	4,777	5.8	ref.	2.56	ref.	6.7	ref.	6.56	ref.	0.8	ref.
ADHD	290	13.4	**2.27 [1.56, 3.30]**	4.44	**1.76 [1.06, 2.47]**	23.2	**7.41 [5.59, 9.82]**	11.13	**4.21 [3.64, 4.78]**	5.5	**7.11 [3.74, 13.51]**
Neither GD nor ADHD	4,394	4.6	ref.	2.23	ref.	5.7	ref.	6.48	ref.	0.6	ref.
GD only	383	20.4	**5.38 [4.03, 7.17]**	6.33	**4.10 [3.49, 4.70]**	9.1	**1.63 [1.12, 2.36]**	7.45	**0.97 [0.52, 1.42]**	2.6	**4.50 [2.15, 9.43]**
ADHD only	225	8.0	1.60 [0.95, 2.69]	3.50	**1.13 [0.45, 1.80]**	35.1	**7.92 [5.79, 10.84]**	10.88	**4.03 [3.38, 4.68]**	4.0	**6.33 [2.81, 14.24]**
Both GD and ADHD	65	32.3	**9.32 [5.37, 16.15]**	7.71	**5.40 [3.53, 7.26]**	33.8	**7.29 [4.25, 12.49]**	11.98	**5.18 [4.10, 6.26]**	10.8	**19.51 [7.90, 48.19]**
Total	5,067	6.3		2.67		7.6		6.82		1.0	
Both GD and ADHD vs. GD only (ref.)			1.73 [0.96, 3.12]		1.30 [−0.66, 3.25]		**4.48 [2.39, 8.40]**		**4.21 [3.05, 5.36]**		**4.34 [1.55, 12.14]**
Both GD and ADHD vs. ADHD only (ref.)			**5.81 [2.84, 11.90]**		**4.27 [2.29, 6.25]**		0.92 [0.51, 1.66]		1.15 [-0.10, 2.39]		**3.08 [1.09, 8.73]**

*ADHD, attention deficit hyperactivity disorder. GD, gaming disorder. Adjusted for age, language, and substance use disorders at wave 1. Significant (p < .05) coefficients are in bold*.

**Figure 2:**
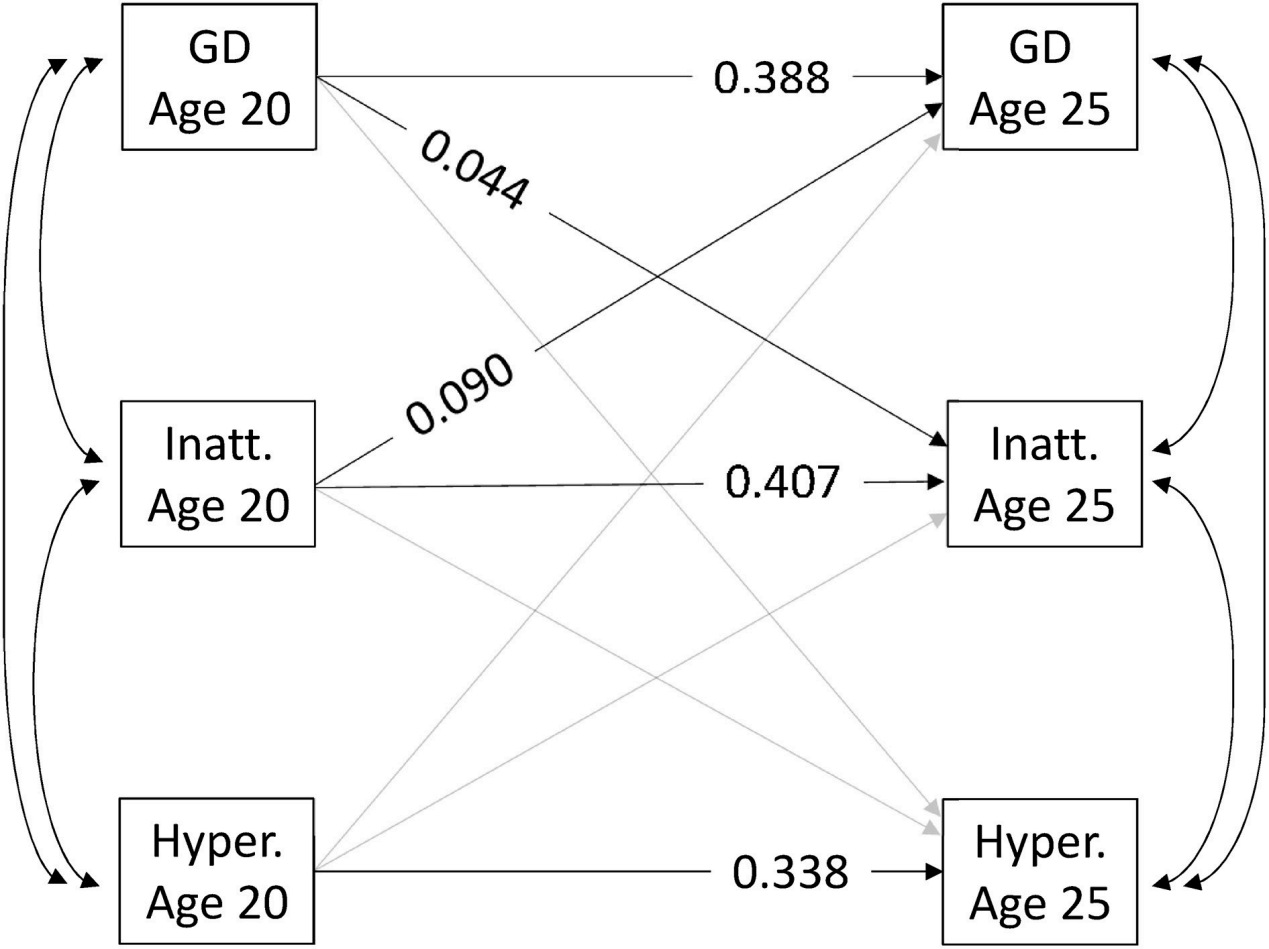
Autoregressive cross-lagged model between continuous measures of gaming disorder and the inattention and hyperactivity subscales of ADHD. GD, gaming disorder; Inatt, inattention; Hyper, hyperactivity. Only significant (*p* < .05) coefficients are shown. Paths in grey were estimated, but were not significant. MLR was the estimator used. Coefficients are standardised beta. Adjusted for age, language, and substance use disorders at wave 1.

As regards longitudinal associations between ADHD subscale scores and GD score, the ARCL including the GD score and the ADHD inattention and hyperactivity subscales only showed significant (notably bidirectional; see Figure 2) associations between GD score and the ADHD inattention subscale (standardized Beta from inattention at age 20 to GD score at age 25: 0.090 [0.056, 0.124]; *p* < 0.001; from GD score at age 20 to inattention at age 25: 0.044 [0.016, 0.071]; *p* = 0.002). The ADHD hyperactivity subscale showed no significant longitudinal associations with GD score (standardized Beta from hyperactivity at age 20 to GD score at age 25: −0.025 [−0.054, 0.005]; *p* = 0.102; from GD score at age 20 to hyperactivity at age 25: 0.004 [−0.023, 0.031]; *p* = 0.755).

### Outcomes in Participants With Comorbid GD and ADHD

As shown in Table 3, the prevalence of GD at wave 3 was highest in participants with GD and ADHD at wave 1 (32.3%), followed by those with GD only at wave 1 (20.4%) and then those with ADHD only at wave 1 (8.0%). These still showed GD somewhat more frequent than participants with neither GD nor ADHD at wave 1 (4.6%). Thus, having ADHD only at wave 1 was associated with higher rates of GD in wave 3 compared to participants with neither GD nor ADHD at wave 1 [unadjusted OR = 1.81 [1.10, 3.00]; after adjustment for age, language, and SUDs, the coefficient (OR = 1.60 [0.95, 2.69]) was just below the significance level]. Furthermore, GD at wave 1 was more likely to persist into wave 3 among participants with ADHD and GD at wave 1 than among participants with GD only at wave 1 (the unadjusted coefficient was 1.87 [1.05, 3.32], however, after adjustment for age, language and SUDs the resulting coefficient was just below significance: OR = 1.73 [0.96, 3.12]). On the other hand, although GD at wave 1 was associated with new onsets of ADHD in wave 3 (9.1% compared to 5.7% in the reference group: OR = 1.63 [1.12, 2.36]), ADHD was not more persistent in wave 3 among participants with GD and ADHD at wave 1 (33.8%) compared to participants with ADHD only at wave 1 (35.1%; adjusted OR = 0.92 [0.51, 1.66]). Finally, the combination of ADHD and GD in wave 3 was most frequent (10.8%) among participants who already had ADHD and GD in wave 1, but this combination's rate of persistence (10.8%) was not very high.

The participants with a combination of GD and ADHD at wave 1 had the worst scores for all the other outcomes measured (Table 4): highest scores on major depression, lowest scores on mental health and life satisfaction, and highest frequency of poor performance at work or school. Participants with ADHD only at wave 1 had somewhat better outcomes than those with GD and ADHD at wave 1; participants with GD only at wave 1 were better still (although not all the coefficients were significant), and those with neither GD nor ADHD at wave 1 had the most positive other outcomes.

**Table 4:** Scores for major depression, mental health, life satisfaction and poor performance at work/school as a function of gaming disorder and ADHD status at wave 1.

**GD and ADHD at wave 1 (age 20)**	**Outcomes at wave 3 (age 25)**							

	**MD score**	***b*** **[95% CI]**	**SF12 MH score**	***b*** **[95% CI]**	**Life satisfaction**	***b*** **[95% CI]**	**Poor performance at work or school**	**OR [95% CI]**
No GD	8.22	ref.	47.20	ref.	26.22	ref.	1.42	ref.
GD	11.75	**2.06[1.29, 2.82]**	43.73	**−1.66 [−2.58**, **−0.75]**	23.91	**−2.26 [−2.92**, **−1.61]**	1.63	**1.28 [1.03, 1.58]**
No ADHD	8.20	ref.	47.33	ref.	26.22	ref.	1.41	ref.
ADHD	13.94	**2.60 [1.60, 3.60]**	39.76	**−3.72 [−4.93**, **−2.51]**	22.73	**−2.99 [−3.82**, **−2.15]**	1.94	**2.07 [1.61, 2.66]**
Neither GD nor ADHD	7.97	ref.	47.57	ref.	26.38	ref.	1.40	ref.
GD only	10.96	**1.83 [1.02, 2.64]**	44.63	**−1.51 [−2.50**, **−.52]**	24.31	**−2.06 [−2.74**, **−1.37]**	1.54	1.18 [0.94, 1.48]
ADHD only	13.22	**2.25 [1.12, 3.37]**	40.15	**−3.65 [−5.03**, **−2.27]**	23.07	**−2.77 [−3.68**, **−1.86]**	1.88	**1.95 [1.48, 2.57]**
Both GD and ADHD	16.35	**4.97 [2.99, 6.95]**	38.42	**−5.19 [−7.56**, **−2.82]**	21.57	**−4.45 [−6.31**, **−2.60]**	2.15	**2.47 [1.42, 4.29]**
Total	8.53		46.90		26.02		1.44	
Both GD and ADHD vs. GD only (ref.)		**3.14 [1.05, 5.24]**		**−3.68 [−6.21**, **−1.15]**		**−2.40 [−4.36,−0.44]**		**2.10 [1.16, 3.77]**
Both GD and ADHD vs. ADHD only (ref.)		**2.72 [0.50, 4.94]**		−1.54 [−4.23, 1.16]		−1.68 [−3.72,0.36]		1.27 [0.69, 2.31]

*ADHD, attention deficit hyperactivity disorder; GD, gaming disorder; MD, major depression; MH, mental health. Adjusted for age, language, and substance use disorders at wave 1, as well as for the baseline values (wave 1) of the respective measures for MD, SF12 MH, and poor performance at work or school. Baseline scores were not available for life satisfaction. Significant (p < .05) coefficients are in bold*.

## Discussion

This study aimed to re-examine the association between (GD) and attention deficit hyperactivity disorder (ADHD) in a longitudinal sample of young Swiss men. At both measurement points, GD was considerably more frequent (OR wave 1: 3.21 [2.39, 4.32]; OR wave 3: 2.56 [1.86, 3.52]) among participants with ADHD than among those without ADHD. Similarly, ADHD was more frequent among participants with GD than those without GD. These findings are well in line with existing studies showing cross-sectional associations between GD and ADHD (8). Importantly, our study also identified longitudinal associations in both directions: ADHD at age 20 increased the risk of GD at age 25, and GD at age 20 increased the risk of ADHD at age 25. So far, only few studies investigated longitudinal associations (8) between ADHD and GD, and, to the best knowledge of the authors, no study showed yet bidirectional associations between ADHD and GD.

Several theories have been proposed concerning the mechanisms underlying associations between ADHD and gaming. Notably, gaming may optimally stimulate individuals with ADHD by providing an exciting activity with immediate rewards: it may therefore be a way to cope with the symptoms of ADHD. However, because gaming provides exactly what individuals with ADHD may prefer, frequent exposure to such a potent stimulus may in turn reinforce ADHD symptoms (29) and lead to less interest in other important activities like work or school. Gaming may also use up a significant amount of an individual's day, further reducing time spent on other activities that may be less problematic for, or even positively influence, the course of ADHD (27, 31). These effects of the exposure to video gaming may even be amplified if combined with the dysfunctional symptoms of GD, such as preoccupation or obsession with gaming or even withdrawal symptoms when not being able to play. However, it is important to note that none of these potential explanations for the association between GD and ADHD have been backed up with sufficient evidence so far, there is clearly more research needed regarding the mechanism linking GD and ADHD.

### Inattention vs. Hyperactivity

A further finding was that the inattention and hyperactivity subscales of ADHD also showed significant cross-sectional associations with GD. However, if jointly entered in a regression model, only inattention remained significant, indicating that the link between ADHD and GD may be mainly accounted for by this variable. Similarly, the ARCL model using both the continuous ADHD subscales and the GD score showed that the link between ADHD and GD (in both directions) was dominated by the inattention subscale, with longitudinal associations for the hyperactivity subscale being non-significant (and even slightly negative). This finding is consistent with those from an earlier cross-sectionall study (26) of 205 adults, which found that the hyperactivity subscale was not significantly linked with GD. Panagiotidi (26) suggested that a potential explanation for the link between the ADHD inattention subscale and GD was that gaming improved visual attention and therefore individuals with ADHD might use gaming as a form of self-medication for the impairments to their attention. On the other hand, a study of young children (27) found that the hyperactivity subscale was associated more strongly with GD among boys, while the inattention subscale was more strongly associated with GD among girls. However, the fact that this sample was much younger (mean age 5.8 years) and the questionnaires were therefore filled out by their parents, makes these results difficult to compare with ours. Lopez et al. (48) also reported that substance abuse problems, which may share some mechanisms with behavioural addictions, were more frequent in individuals with the combined inattention-and-hyperactivity subtype than in those with the predominantly inattentive subtype. There is certainly more research needed regarding the association of ADHD components with GD.

### Outcomes of Participants With GD and ADHD

The present study tested whether individuals with GD and ADHD at age 20 had worse outcomes at age 25 than individuals with only GD or only ADHD. Our results indicate that GD might have been more persistent (i.e., present in waves 1 and 3) among individuals who also had ADHD at age 20 than among those with only GD at age 20, however, the coefficient in our study was just below significance after adjustment for SUD, indicating that other factors besides ADHD may also influence the persistence of GD. This is in line with similar evidence from the field of SUDs showing that ADHD may have a negative impact on the courses of those disorders, i.e., individuals with ADHD may become addicted more easily and have lower remission rates (15). The present study suggests that this may not only be the case for SUDs but also for outcomes such as GD. However, ADHD was not more persistent among participants with comorbid GD and ADHD at age 20 than among participants with ADHD only at age 20. This indicates that GD may not negatively influence the course of already existing ADHD.

At age 25, participants with both ADHD and GD at age 20 had the worst outcomes on all the other scales measured—SF-12 mental health scale scores, major depression scores, life satisfaction, and poor performance at work or school. Participants who only had ADHD at age 20 had the second-worst outcomes. Participants who only had GD at age 20 had somewhat better outcomes at age 25 than those with only ADHD at age 20. Participants who had neither ADHD nor GD at age 20 had the best other outcomes. However, the differences in other outcomes between participants with GD and ADHD at age 20 and those with only ADHD were relatively small and only significant for major depression scores. However, there were relatively few cases with both GD and ADHD at wave 1.

Nevertheless, our results provide evidence that individuals with GD and ADHD may have worse outcomes than individuals who only have GD or who only have ADHD. They also suggest that GD is more than just a symptom or correlate of ADHD, as it is associated with worse outcomes even in individuals with ADHD. GD should therefore be considered as a potentially serious condition, and individuals with comorbid ADHD and GD may require special consideration.

### Limitations

Our sample consisted only of young Swiss men of a restricted age range. Thus, our results may not be generalizable to other populations. Overall, although coefficients for longitudinal associations between GD and ADHD were significant, they were relatively small. However, they remained relatively unchanged, even when adjusted for potentially confounding variables like SUDs. The instrument used for measuring GD differed somewhat between waves 1 and 3, as the Game Addiction Scale was extended in waves 1 and 2 to assess internet addiction too. This was partly corrected by setting the instrument's score to 0 for participants who played video games less than weekly. Overall, small differences in prevalence rates were in the expected direction (lower prevalence with increasing age), and consistent results indicated that the impact of the differences in wording between the instruments was small. For space reasons, we used the short, six-item screener version of the Adult ADHD Self-Report Scale, consisting of only four items for inattention and two for hyperactivity. Further research using longer ADHD scales, allowing for better differentiation of subtypes, is certainly required.

### Conclusion

The present study adds to existing evidence that GD may be associated with serious negative mental health outcomes by providing evidence that GD and adult ADHD have bidirectional longitudinal associations, i.e., each increases the risk of the other. This also suggests the possibility that the two disorders may reinforce each other, i.e., cause a vicious circle (49): early ADHD may facilitate the development of GD, which in turn may over time worsen ADHD, which may again worsen GD. Furthermore, we showed that these bidirectional associations were due more to the inattention subscale of ADHD than to its hyperactivity subscale, which was not independently associated with GD. Young people with GD and ADHD may have worse outcomes than individuals presenting with only one of the two disorders, and they may therefore need special consideration. Accordingly, people with either ADHD or GD should be screened for the other disorder. Effective treatments for ADHD may prevent the onset of GD (49), for example integrated cognitive behavioural therapy as used in the treatment for ADHD and comorbid SUDs (50). Preventive measures for promoting a more appropriate use of computer games by individuals with present ADHD may be helpful. Individuals with an inattention ADHD subtype may need special attention regarding their gaming activities.

## Author Contributions

SM analysed the data and wrote the paper. GG and JS designed the study. GG, JS, and VG assisted data analysis and commented on earlier versions of the manuscript.

### Conflict of Interest Statement

The authors declare that the research was conducted in the absence of any commercial or financial relationships that could be construed as a potential conflict of interest.
